# Eating disorder and bipolar mental illness through genome wide association studies

**DOI:** 10.1192/j.eurpsy.2024.1153

**Published:** 2024-08-27

**Authors:** B. Abdelmoula, R. Rhaiem, A. Charfi, S. Kotti, I. Masmoudi, Y. Marsaoui, N. Bouayed Abdelmoula

**Affiliations:** Genomics of Signalopathies at the service of Precision Medicine - LR23ES07, Medical University of Sfax, Sfax, Tunisia

## Abstract

**Introduction:**

Besides the role played by environmental factors and their epigenetic influences, scientific researchers showed that the susceptibility to develop an eating disorder among bipolar people is due to genetic factors.

**Objectives:**

To review the genetic factors behind eating disorders, highlight the role of genetics and epigenetics in the comorbidity of bipolar and eating disorders.

**Methods:**

To delineate the role of genetics and epigenetics in eating disorders and bipolar disorders as two related mental illness, we comprehensively reviewed the scientific literature using GWAS (genome wide association studies) catalog databases to find genome-wide association studies carried out on patients with bipolar disorder EFO_0005203 and eating disorder comorbid condition (anorexia nervosa, binge eating, bulimia nervosa) EFO_0005203.

**Results:**

GWAS of eating disorders were found in 33 studies with 324 associations whereas those of bipolar disorder were found in 114 studies with 1469 associations. GWAS of eating disorders within bipolar disorders revealed 182 and 134 associations, as well as 10 and 8 publications respectively. Only anorexia nervosa and binge eating were studied in association with bipolar disorders. The genetic variants were protein coding genes (CUBN, FAM228B, FXR1, etc.), non-coding RNA genes (SOX2-OT, MMADHC-DT, etc.), and pseudo-genes (RNU1-23P, CACYBPP2, etc.).

**Conclusions:**

About 300 genetic variants are associated with eating disorder as a comorbid condition of bipolar disorders. These variants may play a crucial role in the causes and mechanisms of eating disorders and should be more investigated towards more precise clinical and genetic entities.

**Disclosure of Interest:**

None Declared

